# Plasma miR-195-5p predicts the severity of Covid-19 in hospitalized patients

**DOI:** 10.1038/s41598-023-40754-w

**Published:** 2023-08-23

**Authors:** Alexandra Ioana Moatar, Aimee Rodica Chis, Mirabela Romanescu, Paula-Diana Ciordas, Diana Nitusca, Catalin Marian, Cristian Oancea, Ioan-Ovidiu Sirbu

**Affiliations:** 1 Department of Biochemistry and Pharmacology, Discipline of Biochemistry, University of Medicine and Pharmacy “Victor Babes”, E Murgu Square no.2, 300041 Timisoara, Romania; 2 Doctoral School, University of Medicine and Pharmacy “Victor Babes”, E Murgu Square no.2, 300041 Timisoara, Romania; 3 Center for Complex Network Science, University of Medicine and Pharmacy “Victor Babes”, E Murgu Square no.2, 300041 Timisoara, Romania; 4 Department of Infectious Diseases, Discipline of Pulmonology, University of Medicine and Pharmacy “Victor Babes”, E. Murgu Square no.2, 300041 Timisoara, Romania; 5 Center for Research and Innovation in Precision Medicine of Respiratory Diseases, “Victor Babes” University of Medicine and Pharmacy Timisoara, E. Murgu Square 2, 300041 Timisoara, Romania; 6 Timisoara Institute of Complex Systems, 18 Vasile Lucaciu Str, 300044 Timisoara, Romania

**Keywords:** Biomarkers, Viral infection

## Abstract

Predicting the clinical course of Covid-19 is a challenging task, given the multi-systemic character of the disease and the paucity of minimally invasive biomarkers of disease severity. Here, we evaluated the early (first two days post-admission) level of circulating hsa-miR-195-5p (miR-195, a known responder to viral infections and SARS-CoV-2 interactor) in Covid-19 patients and assessed its potential as a biomarker of disease severity. We show that plasma miR-195 correlates with several clinical and paraclinical parameters, and is an excellent discriminator between the severe and mild forms of the disease. Our Gene Ontology analysis of miR-195 targets differentially expressed in Covid-19 indicates a strong impact on cardiac mitochondria homeostasis, suggesting a possible role in long Covid and chronic fatigue syndrome (CFS) syndromes.

## Introduction

MicroRNAs are small non-coding RNAs that modulate gene expression at the post-transcriptional level^1^. The relationship between microRNA and their targets is collectively biunivocal: a microRNA can target multiple RNAs and a target RNA can interact (simultaneously or consecutively) with multiple microRNAs^2^. In humans, it is estimated that microRNAs regulate the expression of over 60% of all coding genes^3^.

Respiratory viral infections associate significant changes in host microRNAs expression, which modulate multiple layers of antiviral defense and could even directly interact with the virus^4–6^. The host microRNA response reflects not only the antiviral mechanisms brought into action but also the type of respiratory virus involved^7,8^. Furthermore, viral proteins have been shown to impair the activity of multiple players involved in miR-mediated translational gene silencing^9^. DNA and RNA viruses like herpes viruses, hepatitis C, or the severe acute respiratory syndrome coronavirus (SARS-CoV-2) could sponge out host microRNAs^10–15^, and their pathogenicity seems to correlate with the number of microRNA target sites in the viral genome.

Whether of viral or host origin, given their outstanding stability in biological fluids^16^, the microRNAs hold the potential to become diagnostic and prognostic biomarkers in viral infections^17–23^. The host microRNA response to severe SARS-CoV2 infection has been extensively studied and revealed significant microRNA changes in various stages of the Covid-19 disease^24–40^. The data advanced by these studies are largely non-overlapping and, at times, conflicting, which reflects differences in study design, cohort size, disease stage, and the technological platforms used.

Hsa-miR-195-5p (miR-195) is a member of the miR-15 family known for impacting genes regulating mainly cell proliferation and apoptosis^41,42^. Dysregulation of miR-195 is a relatively common response to viral infections, including HIV-1/HIV-2^43,44^, enteroviruses^45^, and SARS-CoV-2^24^. MiR-195 was described as a possible interactor with all members of the Coronavirus families, including SARS-CoV-2^46^. It is still unclear whether this interaction has deleterious effects on viral RNA stability and translation or if it boosts viral replication. By sponging miR-195 in the infected cells, the SARS-CoV-2 virus might deplete both local and circulant miR-195 levels, with a consecutive impact on the host's immune response^11,12^. This hypothesis becomes even more plausible given that SARS-CoV-2 viral RNA can reach up to 50% of the total RNA of the infected cells^47^.

Given that the ACE2 (angiotensin-converting enzyme 2) receptor is expressed quasi-ubiquitously, Covid-19 manifests as a multi-organ disease involving the lung (the primary target), heart, brain, liver, kidney, intestine, and reproductive tract. Whether acting upon pre-existing organ conditions or disease-free organs, SARS-CoV-2 aggression triggers inflammation, hypoxia, thrombosis, cytokine storm, and sepsis, phenomena driven by signaling, metabolic, genetic, and epigenetic mechanisms^48^. Of note, experimental and observational data place miR-195 in basically all these mechanisms: heart failure and heart ischemia associate augmented miR-195 plasma levels^49,50^, miR-195 protects against multi-organ injury in sepsis^51^, against ischemic and hemorrhagic stroke^52^, acute kidney injury^53^, and thrombosis and endothelial dysfunction^54^. Last but not least, miR-195 is a modulator of two of the main components of SARS-CoV-2 induced cytokine storm, Il-6 and Il-8^55^.

Here we quantified the plasma miR-195 at the onset of Covid-19 disease, analyzed its correlation with clinical and paraclinical parameters of Covid-19 patients, and estimated its transcriptome impact in lung, heart, lymphatic nodes, liver, and kidneys. We show that miR-195 is downregulated in Covid-19 plasma samples, inversely correlates with SARS-CoV-2 RNAemia, efficiently discriminates between severe and mild forms of Covid-19, and impacts the mitochondrial respiration in cardiac muscle.

## Results

### Patients' characteristics

The main characteristics of Covid-19 patients are shown in Table 1 and Supplementary File 1. There are significant differences between the severe and mild Covid-19 cohorts regarding age (P = 0.0201), the incidence of cardiovascular (P = 0.0015), and oncologic pathology (P = 0.0037). Overall, the control patients are older (p = 0.034) and with a lower incidence of oncologic pathology than Covid-19-all patients.

**Table 1:** Demographics data of the Covid-19 patients and controls included in the study.

	Controls (N = 29)	Covid-19 patients (all, N = 89)	Covid-19 patients (severe, N = 27)	Covid-19 patients (mild, N = 62)	P value (severe vs. mild)
Age (Mean + SD)	63.45 ± 8.87	58.12 ± 16.34	66.63 ± 16.74	54.42 ± 14.83	0.0021*
Gender (M/F)	0.93	1.225	1.7	1.07	0.323
Hospitalization days (Mean + SD)	–	13.44 ± 8.26	15.19 ± 12.39	12.68 ± 5.57	0.857**
Risk factors					
Hypertension (%)	51.72	48.31	62.96	41.94	0.069
Obesity (%)	6.89	15.73	7.41	19.35	0.156
Diabetes (%)	6.89	19.10	14.81	20.97	0.4965
Cardiovascular pathology (%)	37.93	41.57	66.67	30.65	0.0015
Oncologic pathology (%)	3.45	11.24	25.93	4.84	0.0037
Medication					
Kaletra	–	14.61	7.41	17.74	0.2041
Plaquenil	–	1.12	3.7	0	0.1285
Tocilizumab	–	0	0	0	–
Corticosteroids	–	86.52	74.07	91.94	0.0232
Anticoagulant	–	98.88	100	98.39	0.5093
Antibiotics	–	80.90	81.48	80.65	0.9283

*Two-tailed unpaired t-test with Welch correction

**Two-tailed Mann Whitney test; all other P values are calculated using the two-tailed Z-test.

Except for the C-Reactive Protein (CRP) (P = 0.0348), fibrinogen (P = 0.0029), prothrombin time (TQ) (P = 0.0026), D-dimers (P = 0.0027) and creatinine (P = 0.0165), there are no significant differences between the paraclinical parameters of the patients from severe versus mild cohorts (Table 2). Clinically, the patients in the severe cohort complain more frequently of dyspnea (P = 0.0183) and thoracic pain (P < 0.0001); their thoracic CT images show more often large glass (P = 0.0209) and consolidated (P = 0.0032) opacities accompanied by diffuse infiltrates (P = 0.004). All data depicting the comparison between the two cohorts are available in Supplementary File 2.

**Table 2:** Clinical and paraclinical parameters of severe and mild Covid-19 patients.

	Severe	Mild	P value
	Mean (± SD)	Mean (± SD)	
Temperature (°C)	37.81 ± 1.004	37.54 ± 1.04	0.3078
Pulse (bpm)	80.2 ± 9.58	78.08 ± 29.22	0.6497
Leukocytes (× 10^3^/µL)	8.3 ± 5.78	7.31 ± 4.05	0.5476
Neutrophils (× 10^3^/µL)	6.44 ± 4.92	5.29 ± 3.71	0.3543
Lymphocytes (× 10^3^/µL)	1.28 ± 0.94	1.28 ± 0.67	0.4652
Thrombocytes (10^3^/µl)	237.5 ± 100.8	228.7 ± 78.66	0.7405
Total bilirubin (mg/dL)	0.41 ± 0.15	4.72 ± 26.62	0.2597
Ferritin (µg/L)	1564 ± 2689	685.1 ± 621.8	0.1634
CRP (mg/L)	73.09 ± 75.24	49.14 ± 84.16	0.0348
Fibrinogen (g/L)	22.86 ± 89.58	4.6 ± 1.54	0.0029
TQ (s)	12.05 ± 1.087	12.23 ± 7.08	0.0026
Hemoglobin (g/dl)	14.41 ± 5.269	14.22 ± 3.18	0.5301
Glucose (mg/dl)	131.7 ± 63.53	132.5 ± 67.4	0.809
Creatinine (mg/dl)	0.97 ± 0.33	0.85 ± 0.44	0.0165
ALAT (U/L)	40.44 ± 37.92	38.68 ± 35.07	0.9841
ASAT (U/L)	40.31 ± 35.46	35.96 ± 35.47	0.2272
LDH (U/L)	308.9 ± 169.8	270.5 ± 108.6	0.6946
D-Dimers (µg/ml)	1.818 ± 4.19	0.55 ± 0.54	0.0027
Potassium (mmol/L)	4.241 ± 0.66	4.26 ± 0.54	0.9011*
Sodium (mmol/L)	137.7 ± 3.52	136.6 ± 4.1	0.3185*

*Two-tailed unpaired t-test with Welch correction; all other P values are calculated using the two-tailed Mann Whitney test.

### miR-195 expression

**Figure 1:**
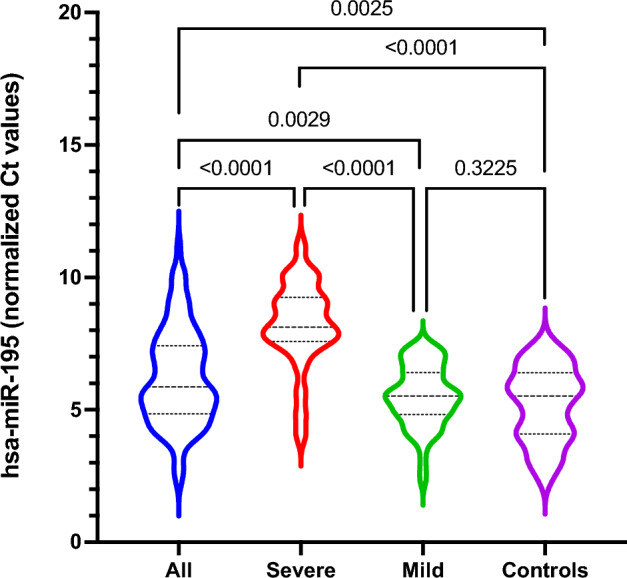
Violin plot showing the normalized (vs. cel-miR-39) plasma miR-195 Ct values in the four cohorts (Covid-All, Covid-Severe, Covid-Mild, Controls). The numbers represent the P values of statistical comparisons (unpaired t-test with Welch correction) between the four cohorts.

Overall, the patients infected with SARS-CoV-2 show a lower plasma level of miR-195 than controls (log_2_FC = − 0.817). In the severe Covid-19 cohort, plasma miR-195 level is significantly reduced (P values < 0.0001) compared to control (log_2_FC = − 2.99), Covid-19 mild (log_2_FC = − 2.695) and Covid-19-all (log_2_FC = − 1.877) cohorts (Fig. 1), while the difference between the mild and the control cohorts (log_2_FC = − 0.3) does not reach statistical significance.

### miR-195 correlations

Next, we analyzed the correlations of plasma miR-195 levels with the clinical and paraclinical characteristics of the patients in the three cohorts. In the Covid-all cohort, miR-195 plasma level is correlated with risk factors, clinical, paraclinical and imagistic signs known for their association to Covid severity: age (r = 0.273, P = 0.0096), cardiovascular pathology (r = 0.234, P = 0.0028), thoracic pain (r = 0.385, P = 0.0003), large consolidated opacities (r = 0.331, P = 0.009) and diffuse infiltrates (r = 0.253, P = 0.048), TQ (r = 0.272, P = 0.0103), D-dimers (r = 0.376, P = 0.0006). Surprisingly, we found a negative correlation with antibiotic (r = -0.223, P = 0.03582) and corticosteroid (r = − 0.282, P = 0.00743) therapy. Of note, plasma miR-195 strongly correlates with all three classifiers for clinical severity: mechanical ventilation (r = 0.543, P < 0.0001), O2 supplementation (r = 0.759, P < 0.0001), and fatal clinical evolution (exitus) (r = 0.258, P = 0.015). Except for O_2_ supplementation, all the other correlations become statistically insignificant upon correlation analysis of the severe cohort (Table 3). The entire correlation analysis data set for Covid-all, Covid-severe, and Covid-mild cohorts is provided in Supplementary File 3.

**Table 3:** Correlation analysis of miR-195 plasma level with patients’ age, plasma D-dimers concentration, thrombin time (TQ), mechanical ventilation, oxygen supplementation and survival outcome.

Correlation coefficient r (P two-tailed)	Covid-all	Covid-severe	Covid-mild
Age	0.2732 (9.58E−03)	− 0.2608 (1.89E−01)	0.1337 (3.00E−01)
D-Dimers	0.3759 (5.89E−04)	− 0.18 (4.11E−01)	0.3937 (2.44E−03)
TQ	0.2723 (1.03E−02)	0.4254 (8.96E−02)	0.1724 (1.84E−01)
Mechanical ventilation*	0.543 (< 1.00E−05)	0.376 (5.36E−02)	–
O_2_ supplementation*	0.759 (< 1.00E−05)	0.6724 (1.2E−04)	–
Exitus*	0.2576 (1.48E−02)	− 0.3387 (8.29E−02)	–

*Point Biserial two-tailed tests; all other values are calculated using two-tailed Spearman tests.

### miR-195 and SARS-CoV-2 RNAemia

We detected traces of SARS-CoV-2 virus (*Orf*, *N*, or *S* gene fragments) in only 24/89 (26.96%) of Covid-all plasma samples, significantly more often in severe versus mild Covid patients (44.44% vs. 19.35%, two-tailed Z test, p = 0.0143). Of the three genes tested, only *N* is significantly different (unpaired T-test with Welch correction) in severe versus mild COVID cases (p = 0.041) and fatal versus non-fatal Covid cases (p = 0.035). In the Covid-all cohort, *N* Ct values are inversely correlated (two-tailed Spearman test with CI = 95%) with miR-195 plasma levels (r = − 0.52, P = 0.011) and fatal outcome (r = − 0.49; p = 0.015); there are no statistically significant correlations between *Orf/S* RNAemia and plasma miR-195 or any of the clinical severity classifiers.

### miR-195 discriminative power

**Figure 2:**
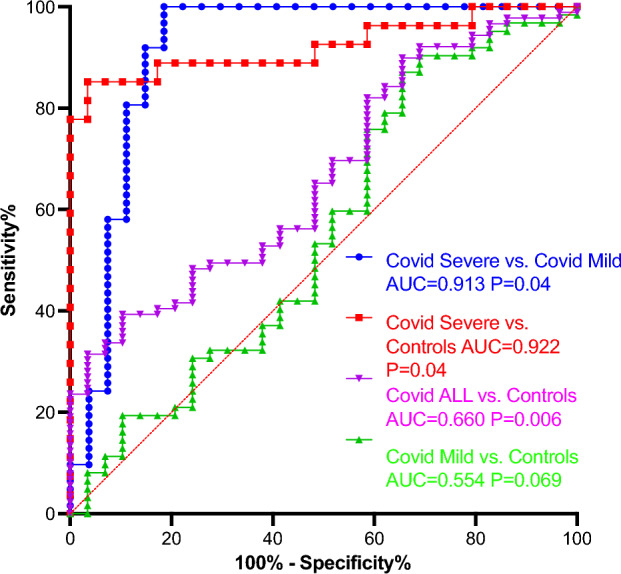
ROC curves (CI calculation with Wilson/Brown method) for the discrimination of severe Covid versus mild Covid, severe Covid versus controls, Covid-all versus controls^81^, and mild Covid versus Controls.

Next, we asked whether miR-195 could discriminate between Covid-19 patients and controls, and between severe and mild Covid-19 patients (Fig. 2, Table 4). AUCs comparisons indicate that miR-195 can distinguish between severe Covid-19 and either controls or mild Covid-19 samples, with AUCs over 0.9.

**Table 4:** Logistic regression/AUC analysis of miR-195 predictive performance in discriminating Covid-all, Covid-mild, Covid-severe versus Control, and Covid-severe versus Covid-mild.

	Covid-all versus Controls	Covid-mild versus Controls	Covid-severe versus Controls	Covid-severe versus Covid-mild
Area	0.6660	0.5545	0.9221	0.9128
Std. Error	0.05504	0.06866	0.04031	0.04452
95% confidence interval	0.5581–0.7739	0.4199–0.6891	0.8431–1.000	0.8255–1.000
P value	0.0074	0.4039	< 0.0001	< 0.0001
Youden index	29.99	21.58	81.74	81.48
Sensitivity %	39.3	87.1	85.19	100
Specificity %	89.7	34.48	96.55	81.48
Likelihood ratio	3.801	1.329	24.7	5.4

The miR-195 ability to discriminate between severe and mild cases surpasses those of any of the biological markers tested: CRP (AUC = 0.6436 l P = 0.0352), fibrinogen (AUC = 0.6973; P = 0.0033), TQ (AUC = 0.6991, P = 0.003), D-dimers (AUC = 0.6605, P = 0.0171).

In all logistic regression models built using combinations of variables for which both correlation with miR-195 and differential expression severe versus mild was found, plasma miR-195 is the only predictive parameter that remains statistically significant (P < 0.05). Of note, all our models have significant discriminative powers (AUC > 0.9, P < 0.0001), with both negative and positive predictive powers above 90% (Supplementary File 4).

### miR-195 role in Covid-19 pathophysiology

In order to understand the role miR-195 plasma changes might play in Covid-19 pathophysiology, we designed a three-step approach: miR-195-5p predicted targets (using the TarPmiR random-forest-based algorithm) were cross-referenced with the list of genes found to be deregulated (adjusted P < 0.05) in the lungs, heart, lymph nodes, liver and kidneys of Covid-19 patients (Supplementary File 5)^56^. Next, the list of differentially expressed miR-195 targets was submitted to STRING Values/Rank functional enrichment analysis; only STRING data sets that correctly identified the tissue of origin (lungs, heart, lymph nodes, liver, or kidneys) were further taken into consideration.

Except for the heart muscle, STRING analysis of miR-195 target differentially expressed genes (DEGs) failed to correctly identify the tissue of origin; this correlates with the well-known abundance of miR-195 in heart tissue (Table 5 and Supplementary File 6). Functional enrichment analysis indicates mitochondria as the target organelle, with a strong impact on oxidative phosphorylation and ATP synthesis. This suggests that miR-195 might play a role in Covid-19 pathophysiology by impacting the myocardium's energy production in response to SARS-CoV-2 infection.

**Table 5:** STRING Rank functional enrichment analysis of miR-195 targets differentially expressed in Covid-19 heart tissue (dataset from Part et al. 2022).

	# Term ID	Term description	Enrichment score	False discovery rate
Tissue expression	BTO:0000862	Heart ventricle	3.05098	1.21E−26
	BTO:0001103	Skeletal muscle	2.27217	1.88E−26
	BTO:0001629	Left ventricle	3.01309	5.58E−24
Subcellular compartment	GOCC:0098798	Mitochondrial protein complex	2.6881	3.57E−40
	GOCC:0005743	Mitochondrial inner membrane	2.56487	2.65E−38
	GOCC:0019866	Organelle inner membrane	2.46486	1.85E−37
Biological process	GO:0046034	ATP metabolic process	2.87214	9.23E−32
	GO:0006119	Oxidative phosphorylation	3.2996	3.39E−29
	GO:0045333	Cellular respiration	3.05963	2.53E−26
KEGG Pathway	CL:13334	Respiratory electron transport, ATP synthesis by chemiosmotic coupling, heat production by uncoupling proteins, and cytochrome complex	3.0603	1.01E−25
	CL:13339	Oxidative phosphorylation	3.35054	1.01E−25
	CL:13336	Oxidative phosphorylation and mitochondrial complex I deficiency	3.24969	1.93E−25

## Discussion

In the present study, we quantified miR-195 in the plasma of hospitalized Covid-19 patients and evaluated its utility as a clinical predictor. Our data indicate that although the miR-195 plasma levels correlated with several of the patients' clinical and paraclinical parameters only in the Covid-all cohort, it is an excellent discriminator between the severe and mild forms of the disease, with an accuracy unmatched by any of the molecular and clinical biomarkers tested in our cohorts.

Predicting the clinical course of SARS-CoV-2 infection is a difficult task, given the multi-systemic character of the disease. The prototypical patient at risk for severe Covid-19 disease is an older diabetic male, obese, with associated chronic cardiovascular and immune pathologies^57^. Correspondingly, multiple blood biomarkers related to enhanced inflammation, cellular fitness, autoimmunity, diabetes mellitus, coagulation, and endothelial dysfunction have been proposed as predictors of Covid-19 severity^58–60^.

Circulating host microRNAs have emerged as powerful predictors of Covid-19 severity; however, these data are largely non-overlapping due to differences in the research methodologies and analytical platforms used^61–64^. We found that miR-195 plasma levels inversely correlate with the severity of the disease. This is in line with previously published data showing the down-regulation of host microRNAs, especially in severe cases of Covid-19. Of note, most of the plasma microRNAs (including miR-195) targeting the SARS-CoV-2 genome are strongly deregulated in severe versus moderate and severe versus asymptomatic patients^65,66^. Farr et al. showed that, together with two other microRNAs (miR-423-5p and miR-23a-3p), miR-195-5p identified and distinguished Covid-19 from Influenza with an accuracy of over 95% but was not a suitable marker for stratifying patients based on Covid-19 disease severity^24^. However, their samples were taken up to 15 days post-admission, while our study was strictly limited to the first two days post-admission. It is plausible that the miR-195 plasma levels are dynamic throughout the course of Covid-19.

SARS-CoV-2 can breach the respiratory epithelial barrier, enter the bloodstream, and spread to extra-pulmonary sites^67^. Detection of SARS-CoV-2 in the plasma of Covid-19 patients is associated with increased clinical severity, representing a significant risk factor for intensive care unit admission, mortality, mechanical ventilation, and multiple organ failure^68–70^. SARS-CoV-2 RNAemia also correlates with plasma Il-6 levels and has been proposed as a predictor of extra-pulmonary involvement and poor prognosis^70–74^. Furthermore, cardiac, pulmonary, and renal damage are more characteristic and important in patients with SARS-CoV-2 RNAemia^75^. MiR-195 has dozens of binding sites in both pathogenic (SARS-CoV-2, SARS-CoV, MERS-Cov) and nonpathogenic (HCoV-OC43, HCoV-229E, HCoV-HKU1) coronaviruses, placing miR-195 in the human host panoply of responses to coronaviruses infection^12,76–78^. Similar to the eastern equine encephalitis virus, the Coronaviruses in general (and SARS-CoV-2 in particular) may have accumulated microRNA binding sites to evade immune detection^10^. Since the complementarity with an RNA target could trigger the exonucleolytic degradation of microRNAs, and given the inverse correlation of plasma miR-195 with *N*-gene Covid-all RNAemia, it is plausible that the low levels of plasma miR-195 found in our study reflect a sponging effect by SARS-CoV-2^11,79,80^. Another possible explanation for the miR-195 decrease relates to the global down-regulation of microRNAs in SARS-CoV-infected cells under endoplasmic reticulum^81^ stress^82,83^. The overall number of microRNA expressed in nasopharyngeal swabs was significantly lower in severe versus controls and versus mild Covid-19 patients^84^.

Our STRING functional enrichment analysis unequivocally links miR-195 to cardiac and muscle tissue and identifies mitochondria and cellular respiration as primary targets of miR-195 deregulation. Cardiomyocytes are specifically enriched in angiotensin-converting enzyme 2 (ACE2), which not only provides a direct link to mitochondrial function regulation but also renders them highly susceptible to infection by SARS-CoV-2^85,86^. Like other RNA viruses, due to distinct 5'- and 3’-UTR mitochondrial localization signals, SARS-CoV-2 hijacks the mitochondria and alters their morphology and bioenergetics dynamics once inside the cell^87–89^. On the other hand, miR-195 has long been known as a mitomiR, a microRNA the homeostasis of which is crucial for mitochondrial function and ATP production in various cells, including cardiomyocytes^90–93^. A low level of miR-195 in SARS-CoV-2 infected cardiac cells might contribute to gene upregulation and imbalanced ROS (reactive oxygen species) production in mitochondria; this phenomenon could explain the hyperinflammatory response in the elderly^94,95^. Low mitochondrial fitness might explain the link between Covid-19 severity and risk factors like age, diabetes mellitus, and associated chronic diseases^96^. Of note, in a rat sepsis model, reduced cardiac expression of miR-195 was linked to multiple mechanisms leading to myocardial injury, including inflammation, apoptosis, and oxidative and endoplasmic reticulum stress^97^.

The impact on mitochondria might also explain the development of chronic fatigue syndrome (CFS) symptoms in a significant number of long-Covid patients. CFS associates metabolic and proteomic changes consistent with an altered Bioenergetic Health Index (BHI) and a significant mitochondria dysfunction in the absence of significant alterations in ventilatory exchanges^98–102^. Furthermore, clinical evolution to long Covid is described more often in patients with plasma SARS-CoV-2 RNAemia detectable in the early stages of the disease^103^.

Our data on miR-195 plasma levels suggest that the myocardial impact of SARS-CoV-2 might be a rather early phenomenon, and we speculate that the switch to a severe course of the disease depends on the ability of the cardiac cells to maintain mitochondrial homeostasis. This would be in line with previously published results, showing myocardial damage in autopsy samples from patients with no clinical signs of cardiac involvement^104^.

Our study has several limitations. First, by restricting our study to hospitalized patients, we have missed both the asymptomatic and the minimally symptomatic patients, and thus, our results have relevance in an intra-hospital setting. Second, we have limited our prospective analysis to the hospital records, thus excluding post-hospitalization evolution (including long Covid development) from the analysis. Third, the patients' compliance with the study was reduced, with implications on the size and characteristics of the cohorts taken into the analysis: severe Covid-19 patients tend to be older, with more cardiovascular and oncologic comorbidities. Last but not least, although we included patients from a single county hospital, we are confident the cohorts analyzed are representative of a broader population, given the large regional addressability of CHIDP.

The present data reflect the status of patients recruited in 2020, during the first pandemic wave, and well before the B1.1.7 variant became dominant in Romania; it is unclear whether other variants (less prone to induce severe cases) would associate similar changes in plasma miR-195 levels. Should the viral sponging theory hold true, we would see a similar shift in plasma miR-195, although it is not clear whether its ability to predict the severity of the disease would be affected.

It is also unclear whether the drop in miR-95 plasma level is a consequence or a player in Covid-19 physiopathology, e.g., by modulating the expression of severity-associated factors like Il-6 and Il-8; since all plasma samples were obtained within 48 h upon hospital admission, before ICU admission, it is conceivable that it might play a role in the onset of severity symptoms.

The identification of early downregulation of miR-195 during (severe) Covid-19 might contribute to a better understanding of the molecular mechanisms involved in the pathology of immune response in viral infections. It might also help to understand the pathways involved in regulating excessive inflammatory responses in many human pathologies, thus paving the way for developing potentially useful treatments.

## Materials and methods

### Design

The study enrolled 89 patients with a nasopharyngeal swab test positive for SARS-Cov-2 admitted to the Clinical Hospital of Infectious Diseases and Pneumophysiology (CHIDP), Timisoara, Romania, during the first pandemic wave of Covid-19 (May to December 2020). The Ethics Committee of the Victor Babes University of Medicine and Pharmacy Timisoara (34/28.07.2020) approved the study protocol, which was caried out in accordance with the Declaration of Helsinki. All patients were informed and provided written consent regarding the therapy and investigations performed, recorded as such in their medical record. Based on their respiratory support requirements, the patients’ disease status was classified as severe if any of the following occurred: non-invasive oxygen supplementation with or without non-invasive ventilation, invasive mechanical ventilation, death, and mild (in the absence of all the severe disease criteria).

### Plasma collection

The blood samples were collected in EDTA (ethylenediaminetetraacetic acid)-coated vacutainers within the first two days upon hospital admission, before or around the onset of Covid-19 therapy (interferon-beta, Kaletra, Tocilizumab, Corticosteroids, Heparin). The control samples were collected from healthy controls recruited before the outbreak of the Covid-19 pandemic (2016–2018). All blood samples were processed (centrifuged at 1500*g* for 10 min) within three hours after collection, and plasma was stored aliquoted at – 80 °C until further use.

### Clinical data collection

The patients' demographic and laboratory data and the clinical variables were collected from the CHIDP electronic medical records and stored anonymized on the Biochemistry Department servers (Supplementary File 1). All clinical and paraclinical variables refer to day of admission.

### miR-195 quantification

The RNA was purified from 200uL of plasma spiked with synthetic cel-miR-39-3p for normalization; we used the miRNeasy Serum/Plasma Kit (Qiagen, catalog no. 217184), and followed the manufacturer's instructions. The quality of the RNA was verified on a Nanodrop 2000 Spectrophotometer; all samples had 260/230 ratios and 280/260 ratios between 1.8 and 2.0.

cDNA was synthesized using the TaqMan™ MicroRNA Reverse Transcription Kit (Applied Biosystems, catalog no. 4366596) starting from 10 ng of total RNA and according to the manufacturer's instructions. Hsa-miR-195-5p and cel-miR-39 RT-PCR amplifications were performed using dedicated Taqman microRNA assays (Applied Biosystems, assays ID 000494 and 000200, respectively). The fold change (FC) in miR-195 was calculated using the ΔΔCt method^105^.

### SARS-CoV-2 detection

The RNA was purified from 200uL of plasma spiked with synthetic cel-miR-39-3p for normalization; we used the miRNeasy Serum/Plasma Kit (Qiagen, catalog no. 217184), and followed the manufacturer's instructions. The quality of the RNA was verified on a Nanodrop 2000 Spectrophotometer; all samples had 260/230 ratios and 280/260 ratios between 1.8 and 2.0.

SARS-CoV-2 virus detection (*Orf*, *N*, and *S* gene fragments) was performed using the TaqMan™ 2019-nCoV Assay Kit v1 (Applied Biosystems, Waltham, MA, USA, catalog no. A47532) according to the manufacturer's instructions. RNase P assay was used as an internal control.

### Statistical analysis

The statistical analysis was performed using Prism 9 for MacOS, Version 9.3.1. Descriptive statistics was used to characterize the demographic, clinical and laboratory data of the patients. Data distribution normality was tested using the Shapiro–Wilk test. Differences between continuous variables data sets were assessed using Student’s *t*-test (if normally distributed) and Mann–Whitney *U* tests (if not normally distributed). Binary variables datasets were compared using the Z test. Correlation analyses were performed using the two-tailed Spearman test (continuous variables) and Point Biserial test (continuous vs. binary variables). The statistical significance of miR-195 plasma changes in control, Covid-19-all (severe + mild cases), Covid-19 mild and Covid-19 severe cohorts was calculated using the unpaired t-test with Welch correction. Receiver operating characteristics (ROC) analyses were performed with the standard parameters in Prism 9, using Wilson/Brown method for confidence interval calculation. For all tests, the threshold of statistical significance is 0.05. All statistical tests are two-tailed.

### miR-195 transcriptome impact evaluation

The prediction of miR-195-5p targets was performed using the TarPmiR random-forest-based algorithm for 3′-UTR, CDS, and 5′-UTR target regions^106^. Only the MiRDB- and Targetscan-validated interactions and the interactions with binding probabilities over 0.9 were included in the analysis (Supplementary File 5).

Transcriptome data (including log_2_FC values) for Covid-19 lung, heart, lymph nodes, liver, and kidney were retrieved from Park et al. and cross-referenced to the miR-195 target genes list, then submitted to STRING for Values/Rank functional enrichment analysis (Supplementary File 6)^107^.

### Supplementary Information

Supplementary Information 1.

Supplementary Information 2.

Supplementary Information 3.

Supplementary Information 4.

Supplementary Information 5.

Supplementary Information 6.

Supplementary Information 7.

## Data Availability

The datasets used and/or analysed during the current study are available from the corresponding author on reasonable request.
