# Supplementary material for: Plasma miR-195-5p predicts the severity of Covid-19 in hospitalized patients
Source: Sci Rep. 2023 Aug 23;13:13806. doi: 10.1038/s41598-023-40754-w (PMC10447562; doi:10.1038/s41598-023-40754-w)
Supplement: Supplementary file 7 — Supplementary Information 7. [file 41598_2023_40754_MOESM7_ESM.docx]

**The list of Supplementary Files, including title and legend for each File.**

**Supplementary File 1**. Clinical, paraclinical data and adjusted miR-195 values of the patients included in the study

Legend: CRP – C Reactive Protein; TQ - prothrombin time, ALAT – Alanine Aminotransferase; ASAT – Aspartate Aminotransferase; LDH – Lactate Dehidrogenase

**Supplementary File 2.** Comparisons of Sever-Covid and Mild-Covid cohorts

Legend: CRP – C Reactive Protein; TQ - prothrombin time, ALAT – Alanine Aminotransferase; ASAT – Aspartate Aminotransferase; LDH – Lactate Dehidrogenase; P values below 0.05 threshold are marked in red.

**Supplementary File 3.** Correlations of adjusted miR-195 with clinical and paraclinical parameters.

Legend: CRP – C Reactive Protein; TQ - prothrombin time, ALAT – Alanine Aminotransferase; ASAT – Aspartate Aminotransferase; LDH – Lactate Dehidrogenase; P values below 0.05 threshold are marked in red.

**Supplementary File 4.** Multiple Logistic Regression Analyses using severity as dependent nominal variable.

Legend: P values below 0.05 threshold are marked in red.

**Supplementary File 5.** miRWalk3.0 predictions of miR-195 targets (3’-UTR, CDS, and 5’UTR).

**Supplementary File 6.** STRING analyses of miR-195 targets found differentially expressed (in Park et all. data set) in heart, lung, lymph nodes, liver, and kidney
